# Optimal Low Temperature and Chilling Period for Both Summer and Winter Diapause Development in *Pieris melete*: Based on a Similar Mechanism

**DOI:** 10.1371/journal.pone.0056404

**Published:** 2013-02-18

**Authors:** HaiJun Xiao, ShaoHui Wu, Chao Chen, FangSen Xue

**Affiliations:** 1 Key Laboratory of Crop Physiology, Ecology and Genetic Breeding, Jiangxi Province, Institute of Entomology, Jiangxi Agricultural University, Nanchang, China; 2 State Key Laboratory for Biology of Plant Diseases and Insect Pests, Institute of Plant Protection, Chinese Academy of Agricultural Sciences, Beijing, China; 3 Department of Entomology, Virginia Polytechnic Institute and State University, Blacksburg, Virginia, United States of America; University of Lausanne, Switzerland

## Abstract

The cabbage butterfly, *Pieris melete* hibernates and aestivates as a diapausing pupa. We present evidence that the optimum of low temperature and optimal chilling periods for both summer and winter diapause development are based on a similar mechanism. Summer or winter diapausing pupae were exposed to different low temperatures of 1, 5, 10 or 15°C for different chilling periods (ranging from 30 to 120 d) or chilling treatments started at different stages of diapause, and were then transferred to 20°C, LD12.5∶11.5 to terminate diapause. Chilling temperature and duration had a significant effect on the development of aestivating and hibernating pupae. The durations of diapause for both aestivating and hibernating pupae were significantly shorter when they were exposed to low temperatures of 1, 5 or 10°C for 50 or 60 days, suggesting that the optimum chilling temperatures for diapause development were between 1 and 10°C and the required optimal chilling period was about 50–60 days. Eighty days of chilling was efficient for the completion of both summer and winter diapause. When chilling periods were ≥90 days, the durations of summer and winter diapause were significantly lengthened; however, the adult emergence was more synchronous. The adaptive significance of a similar mechanism on summer and winter diapause development is discussed.

## Introduction

Diapause is one of the primary mechanism whereby insects of temperate zones synchronize their life cycles with local seasonal changes [1]. Diapausing insects experience a graded series of physiologically distinct developmental stages including diapause induction, preparation, initiation, maintenance, termination, and sometimes post-diapause quiescence [2]. Many temperate-zone insects are known to terminate winter diapause by mid-winter. The insects have the potential to resume morphogenesis, and are then able to respond to environmental stimuli and restart its post-diapause development. However, insects may remain in a state of post-diapause quiescence because post-diapause development and morphogenesis are inhibited by low temperatures [3], [4]. On the contrary, a suitable period of exposure to low temperatures usually accelerate the development of diapause [5]. The chilling temperature and length of the cold period required for diapause termination varied among populations within a species and among individuals within a population. Exposure to low temperatures may be a prerequisite for the completion of winter diapause in some species. Increasing evidence shows that diapause completion progresses well at intermediate or high temperatures, and sometimes diapause termination is even stimulated by high or increasing temperatures [3], [6]–[8]. Low temperatures are also important in winter diapause, as they (1) conserve metabolic reserves, (2) prevent resumption of post-diapause morphogenesis and thus synchronize the life-cycle, (3) represent contrast to the later increase in temperature [9]. The influence of low temperature on summer diapause termination has so far only been tested in the onion maggot, *Delia antiqua*, and in the cabbage armyworm, *Mamestra brassicae* [10], [11], perhaps because in many cases summer diapause is terminated in the field without experiencing low temperatures. However, a comparison of the response to low temperature between summer and winter diapause may provide new insight into understanding the mechanism of diapause development.

The cabbage butterfly *Pieris melete*, a serious pest of crucifers in the mountain areas of the Jiangxi Province, P. R. China, undergoes summer and winter diapause responding to relatively long (>13 h) and short (<12 h) day-length, respectively [12]. We are interested in testing the extraordinary flexibility of *P. melete* to the local seasonal environmental challenge, and a series of experiments were conducted under laboratory and natural conditions to obtain insight into the mechanisms of the clock, time measurement and influence of environmental stimuli on induction and intensity of summer and winter diapause in this butterfly [13]–[17]. Previous studies showed that summer diapause development of this insect was delayed at high temperatures, and enhanced at low temperatures, indicating that the optimum temperature of summer diapause development is relatively low. In addition, low temperatures enhanced winter diapause development and termination [12]. However, the mechanism of optimum temperature and minimum period of exposure to chilling conditions required to complete diapause has not been determined. Knowledge of these diapause characteristics would not only help to better understand the ecology of *P. melete*, but also have a potential practical significance in increasing the efficiency of forecasting and managing this pest. It is important to examine the role of temperature in the control of diapause development to obtain basic information for constructing an efficient control technique. In the present study, we investigated in detail on the optimal chilling temperature treatment and period of low temperature exposure for the development of summer and winter diapause in this butterfly.

## Results

### Effect of Chilling on Summer Diapause Development

**Figure 1:**
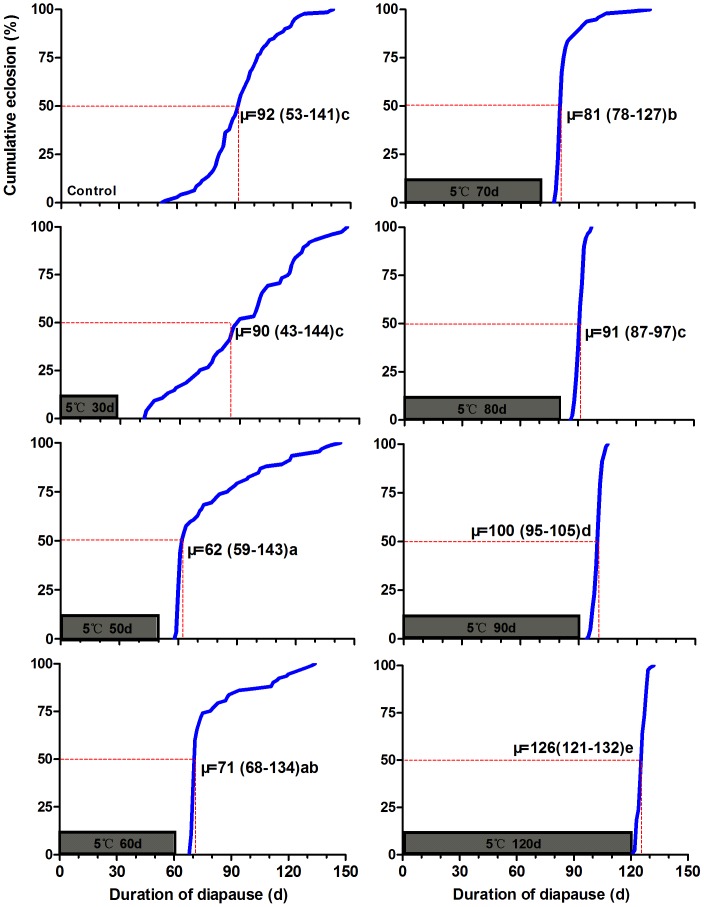
Cumulative percentage of butterfly eclosion in summer diapausing pupae of *P. melete*. The summer diapausing pupae were transferred to LD12.5∶11.5 at 20°C after exposure to 5°C and DD for different days. The hatched bar indicates the period of cold exposure under constant darkness. Data were shown as the median (minimum value–maximal value); values followed by different letters are significantly different by Bonferroni test (P<0.05).

In the control treatment without low temperature chilling, summer diapausing pupae were kept constantly under an intermediate day-length of LD 12.5∶11.5 (light 12.5 h: dark 11.5 h) at 20°C. Under this condition, the first butterfly emerged after 53 d and the last one after 141 d; the pupal period for 50% adult emergence was 92 d (Figure 1, Control). After exposure to 5°C and DD (continuous darkness) for different days, summer diapausing pupae of *P. melete* were transferred to LD12.5∶11.5 and 20°C to eclose into adults (Figure 1). Significant differences in diapause duration were found for summer diapausing pupae chilled at 5°C (Kruskal–Wallis tests, χ^2^ = 352.420, d.f. = 7, *P* = 0.0001). Diapausing pupae that received the 50 d chilling treatment showed the shortest duration of diapause. For diapausing pupae chilled for 80 d, adult emergence was synchronized within 11 d (Figure 1). The average ‘time-to-adult’ was about the same period for eclosion of non-diapausing pupae. By contrast, for diapausing pupae chilled for 90 or 120 d, the duration of diapause was lengthened to 15 and 38 d, respectively (Bonferroni test: *P* = 0.04 between 90 d of chilling and control, *P* = 0.001 between 120 d of chilling and control) (Figure 1).

Effect of chilling temperature and duration on diapause termination and post-diapause development was presented in Table 1. There were no significant differences in diapause development between the control and 30 d of chilling treatment at 1, 5, 10 and 15°C (Bonferroni test: *P* = (0.239–1.000) between 30 d of chilling and control). However, there were significant differences in diapause development among different chilling temperatures when chilling duration was lengthened to 50, 60, 90 and 120 d (Bonferroni test: *P* = 0.0001 between 50 or 60 d chilling and control). The duration of diapause were relatively shorter at 1, 5 and 10°C when the diapausing pupae were exposed to the chilling treatment for 50 or 60 d, under which the duration of diapause was significantly shorter than that of pupae chilled for 30, 90 or 120 d chilling (Table 1, Figure 1, S1 and S2). For chilling treatments at 10°C and 15°C, some individuals emerged into adults during the chilling period before being transferred to 20°C and adult emergence was not synchronized. This suggests that 10 and 15°C falls into the range of temperatures that enable morphological and post-diapause development (Figure S2, S3).

**Table 1:** Effects of low temperature chilling on diapause termination and post-diapause development in aestivating pupae of *P. melete*.

	1°C	5°C	10°C	15°C	Kruskal-Wallis test
Control	92 (53–141) (140) b	92 (53–141) (140) bc	92 (53–141) (140) bc	92 (53–141) (140) a	
30 d	94 (42–144) (70) b A	90 (43–144) (75) b A	89 (44–140) (90) b A	95 (47–144) (57) a A	χ^2^ = 1.023 d.f. = 3 *P* = 0.7955>0.05
50 d	61 (56–135) (86) a A	62 (59–143) A (92) a A	64 (60–136) (88) a A	82 (57–136) (92) a B	χ^2^ = 77.955 d.f. = 3 *P* = 0.0001<0.01
60 d	70 (68–99) (75) a A	71 (68–134) (93) a A	71 (69–126) (79) a A	81 (64–140) (65) a B	χ^2^ = 42.520 d.f. = 3 *P* = 0.0001<0.01
90 d	100 (97–104) (81) c B	100 (95–105) (79) c B	96 (93–109) (89) c A	96 (58–117) (83) ab A	χ^2^ = 93.822 d.f. = 3 *P* = 0.0001<0.01
120 d	129 (127–133) C (83) d C	126 (121–132) (87) d C	121 (92–130) (100) d B	100 (59–132) (70) b A	χ^2^ = 237.480 d.f. = 3 *P* = 0.0001<0.01
Kruskal-Wallis test	χ^2^ = 341.120 d.f. = 5 *p* = 0.0001<0.01	χ^2^ = 262.451 d.f. = 5 *p* = 0.0001<0.01	χ^2^ = 288.310 d.f. = 5 *p* = 0.0001<0.01	χ^2^ = 27.206 d.f. = 5 *p* = 0.0001<0.01	

Data were shown as the median (minimum value–maximal value)(pupae number, dead pupae were not included), value fallowed by the different capital letter within a row, lowercase letter within a column are significant different by Kruskal-Wallis test followed a Bonferroni multiple test (*P*<0.05).

### Effect of Chilling on Summer Diapause Termination in the Later Phase of Diapause Development

**Figure 2:**
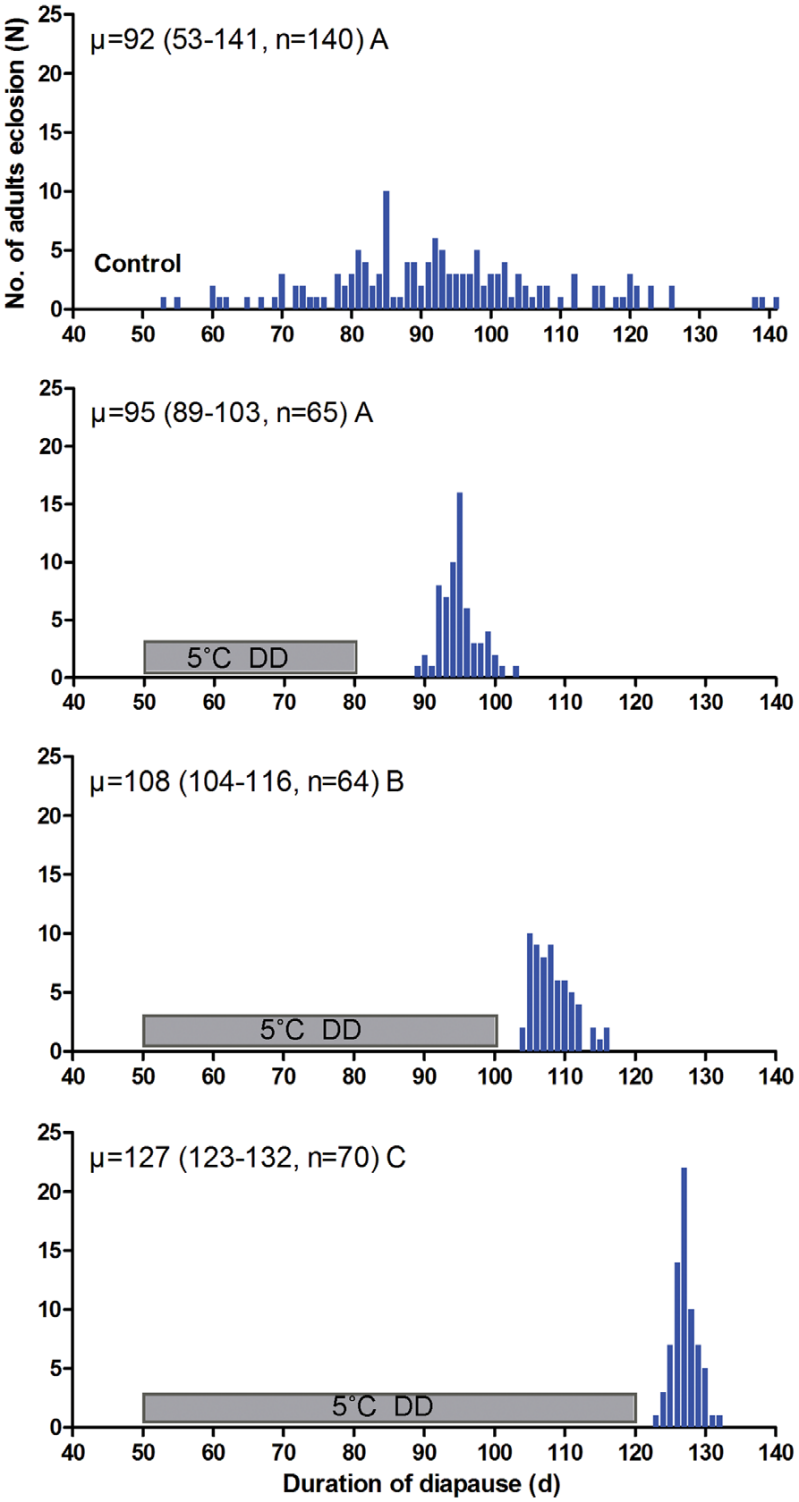
Frequency distribution of adult eclosion in summer diapausing pupae of *P. melete.* The summer diapausing pupae were transferred to LD12.5∶11.5 at 20°C after exposure to 5°C and DD for different days. The hatched bar indicates the period of cold exposure. Values followed by different letters are significantly different by Kruskal-Wallis test and Bonferroni multiple comparison (χ^2^ = 224.312, d.f. = 3, *P* = 0.0001<0.01).

Diapause termination in pupae was prolonged at 5°C in the later phase of diapause development; the average ‘time-to-adult’ was lengthened as chilling period increased. In the later phase of summer diapause development, for pupae chilled at 5°C for 30 d (i.e. from day 50 after pupation to day 80), the average ‘time-to-adult’ was 95 d. The ‘time-to-adult’ was 108 d for summer diapausing pupae chilled at 5°C from day 50 after pupation to day 100, and 128 d for 70 d of chilling exposure starting from day 50 after pupation to day 120. However, the adult emergence was synchronized considerably within 14 d, compared to the control without chilling treatment, for which adults emerged within 88 d. Thus, in the latter phase (>80 d), by contrast, diapause development was delayed in proportion to the duration of chilling (Figure 2).

### Effect of Chilling on Winter Diapause Development

Over-wintering pupae were transferred to low temperature chilling of 1, 5, 10 and 15°C for periods of 30 to 120 d. The duration of diapause was significantly affected by the chilling temperature and the chilling duration (Kruskal-Wallis test: 30 d: χ^2^ = 22.061, d.f. = 3, *P* = 0.0001<0.01; 50 d: χ^2^ = 68.475, d.f. = 3, *P* = 0.0001<0.01; 80 d: χ^2^ = 112.460, d.f. = 3, *P* = 0.0001<0.01; 90 d: χ^2^ = 99.474, d.f. = 3, *P* = 0.0001<0.01; 120 d: χ^2^ = 138.112, d.f. = 3, *P* = 0.0001<0.01) except for 60 d chilling (60 d: χ^2^ = 1.437, d.f. = 3, *P* = 0.1573>0.05) (Table 2).

**Table 2:** Effects of low temperature chilling on diapause termination and post diapause development in hibernating pupae of *P. melete*.

	1°C	5°C	10°C	15°C	Kruskal-Wallis test
Control	93 (45–130) (119) b	93 (45–130) (119) c	93 (45–130) (119) cd	93 (45–130) (119) c	
30 d	71 (42–117) (52) a A	89 (45–135) (73) c B	85 (57–121) (53) b B	88 (49–128) (53) bc B	χ^2^ = 22.061 d.f. = 3 *P* = 0.0001<0.01
50 d	65 (62–114) (51) a A	80 (60–134) (74) b B	77 (68–115) (52) ab B	83 (61–124) (53) b B	χ^2^ = 68.475 d.f. = 3 *P* = 0.0001<0.01
60 d	73 (71–77) (53) a A	71 (68–133) (90) a A	74 (66–112) (50) a A	74 (61–117) (50) a A	χ^2^ = 1.437 d.f. = 3 *P* = 0.1573>0.05
80 d	90 (89–92) (53) b B	95 (91–97) (77) c C	89 (82–95) (51) bc B	85 (51–114) (52) ab A	χ^2^ = 112.460 d.f. = 3 *P* = 0.0001<0.01
90 d	101 (100–103) (53) c C	101 (97–105) (78) d C	96 (92–103) (54) d B	87 (51–106) (52) ab A	χ^2^ = 99.474 d.f. = 3 *P* = 0.0001<0.01
120 d	130 (128–133) (55) d B	129 (124–132) (80) e B	127 (104–133) (55) e B	86 (57–119) (55) bc A	χ^2^ = 138.112 d.f. = 3 *P* = 0.0001<0.01
Kruskal-Wallistest	χ2 = 295.918 d.f. = 6 p = 0.0001<0.01	χ2 = 328.160 d.f. = 6 p = 0.0001<0.01	χ2 = 240.232 d.f. = 6 p = 0.0001<0.01	χ2 = 57.369 d.f. = 6 p = 0.0001<0.01	

Data were shown as the median (minimum value–maximal value) (pupae number, dead pupae were not included), value fallowed by the different capital letter within a row, lowercase letter within a column are significant different by Kruskal-Wallis test followed a Bonferroni multiple test (*P*<0.05).

Chilling at 1°C differed among exposing periods ranging from 30 to 120 d, Chilling periods of 30, 50 and 60 d significantly facilitated diapause development, and the ‘time-to-adult’ was at least 20 d shorter than that of the control treatment. However, when the chilling period was 90 d or longer, diapause development was delayed significantly (Kruskal-Wallis test, 1°C: χ^2^ = 295.918, d.f. = 6, *P* = 0.0001<0.01, Table 2, Figure S4). Diapause durations of hibernating pupae chilled at 5, 10 and 15°C differed significantly for different periods of chilling ranging from 30 to 120 d, and 60 d of chilling significantly accelerated the development of diapause by shorten the duration of ‘time-to-adult’ (Kruskal-Wallis test: 5°C:χ^2^ = 328.160, d.f. = 6, *P* = 0.0001<0.01;10°C:χ^2^ = 240.232, d.f. = 6, *P* = 0.0001<0.01;15°C:χ^2^ = 57.369, d.f. = 6, *P* = 0.0001<0.01, Table 2, Figure S5, S6, S7). For pupae chilled under 1°C for 60 d, adult emergence was synchronized within 7 d. This indicates that 60 d of chilling at 1°C was sufficient for winter diapause completion (Table 2, Figure S4). After being chilled for 80 d at 5 and 10°C, butterfly eclosion was also synchronized within 7 d and 13 d, respectively. Which suggest that 80 d at 5 and 10°C was enough for winter diapause completion (Figure S5, S6). After being chilled at 15°C for various days, adult emergence varied in different treatments. Some adults emerged 104 d after pupation at 10°C, and 51 d at 15°C, and all individuals terminated diapause within 120 d at 15°C, which were faster than the control (Figure S6, S7).

### Effect of Chilling on Winter Diapause Termination in the Later Phase of Diapause Development

**Figure 3:**
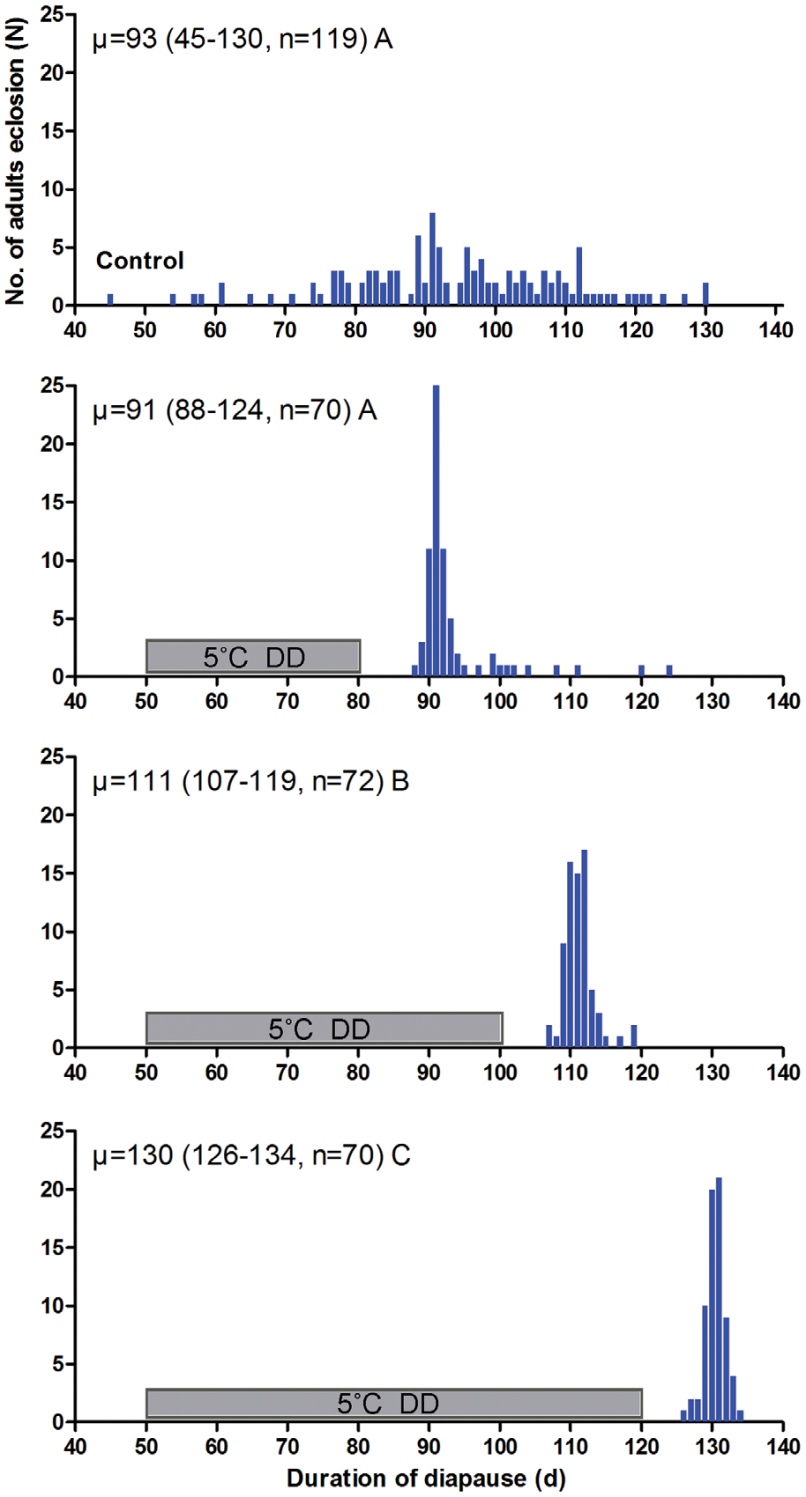
Frequency distribution of adult eclosion in winter diapausing pupae of *P. melete.* The winter diapausing pupae were transferred to LD12.5∶11.5 at 20°C after exposure to 5°C and DD for different days. The hatched bar indicates the period of cold exposure. Values followed by different letters are significantly different by Kruskal-Wallis test and Bonferroni multiple comparison (χ2 = 212.204,d.f. = 3,P = 0.0001<0.01).

Over-wintering pupae were kept constantly at 20°C and LD12.5∶11.5. Adult eclosion began on day 45 and peaked on day 91, and all individuals emerged by day 130. When pupae were chilled at 5°C from day 50 to day 80, the peak of adult eclosion also occurred on day 91. However, more than 85% adult emergence was synchronized within 10 d. When chilling occurred in the later phase of diapause from day 50 to day 100 or from day 50 to day120, pupal development was delayed, and the ‘time-to-adult’ was 111 d and 130 d, respectively (Figure 3).

## Discussion

Summer and winter diapause generally requires distinct stimuli for termination. For a large number of insects in temperate regions, a period of low temperature accelerates the development of diapause, and the length of the chilling period required for the termination of diapause varies among individuals within a population and among populations within the same species [3], [5]. In some insects, diapause development cannot be completed under constant diapause-inducing conditions and lower temperature is required for the completion of diapause, therefore, few or no individual complete diapause under constant diapause-inducing conditions [18]–[21]. In many insects, a chilling treatment shows significant influence on diapause termination, but is not a prerequisite for the completion of diapause, and a longer period of chilling generally facilitates the synchronization of diapause completion [22]–[29]. In the present study, both summer and winter diapause development in *P. melete* could be completed under diapause-inducing conditions without chilling, however, the adult eclosion time was extended over a longer period (88 d for summer diapause; 85 d for winter diapause), significantly longer than those with 80 d of chilling at 5°C (11 d for summer diapause; 8 d for winter diapause). This result suggests that a longer period of chilling may be required for synchronizing the post-diapause development, and thus results in synchronous emergence patterns.

A general model with two different phases during insect diapause was proposed by Koštál (2006) [2]. Diapause development progresses over a wide range of temperatures, and the optimum temperature for diapause development differs in each species [8], [22], [23], [30]–[32]. In the cabbage butterfly *P. melete*, 80 d of chilling resulted in synchronized adult emergence, indicating that the treatment of 80 d at low temperatures was enough for both summer and winter diapause completion. However, when the chilling period was 90 d or longer, the durations of summer and winter diapause were significantly lengthened. Therefore, summer and winter diapause is also comprised of two different phases showing different responses to low temperature chilling, with the watershed occurring at around day 80. In the first phase (before day 80), low temperature chilling accelerated the development of diapause. In the other phase (after 80 d), low temperatures chilling delayed the development of diapause and adjusted the synchronization of adult emergence.

As aforementioned in introduction, the influence of lower temperatures on both summer and winter diapause development has been examined in the onion maggot, *D. antiqua* [10] and the cabbage armyworm, *M. brassicae* [11]. In *D. antique*, 16°C was the optimal temperature for completion of summer diapause in which diapause was completed within 5 d, whereas 5 d of chilling at 2°C only resulted in 40% individuals to terminate diapause [10]. Winter diapause in *D. antique* was completed under constant diapause-inducing conditions of 15°C and short day-length of LD12∶12 without chilling. The chilling treatments at 5.6°C not only had no accelerating effect on winter diapause development, but also delayed it [8]. In the cabbage armyworm, *M. brassicae*, the responses of summer and winter diapause to low temperature chilling were quite different. Winter diapause cannot be completed at 20°C for 5 months without chilling; therefore, low temperature chilling is indispensable for winter diapause development. However, summer diapause development is independent of low temperature chilling. The chilling period for winter diapause termination was about 4 months, whereas 1 month was sufficient for summer diapause termination [11]. In the cabbage butterfly *P. melete*, the present study showed that the optimum chilling temperatures for both summer and winter diapause development were between 1 and 10°C, the required optimal chilling periods were 50–60 d, and 80 d of chilling was enough for both summer and winter diapause completion. To our best knowledge, this is the first report that the influence of chilling on both summer and winter diapause development is based on a similar mechanism.

For pupae that experienced a suitable period of low temperature chilling, the adult eclosion time extended over occurred within a shorter period (about 10 d) than the control treatment (more than 2 months). In the field, this thermal response for diapause development mechanism plays a very important role in the phenology of *P.melete*. In temperate regions, summer diapause completion occurs under changing environmental conditions with first increasing day lengths and fluctuating temperatures in spring-summer, followed by decreasing day lengths and fluctuating temperatures in summer–autumn; and winter diapause, vice-versa. Therefore, the mechanism of prolonged adults emergence for aestivating pupae without chilling, ensures the aestivating individuals are able to synchronized the autumn generation with the growth season of the local host plants [12]. In the southern area of China, the host plants of *P. melete* are harvested within a short period of time (before mid-May). Therefore, the synchronized hibernating individuals’ eclosion of the overwintered generation within this limited time guarantees the availability of food resources to the next generation.

Time needed for diapause termination and post-diapause development rates can be used as an indicator to predict the time of adult emergence. By improving our understanding of the eco-physiological processes underlying the mechanisms of summer and winter diapause, the current study may improve the application of temperature regimes in the forecasting out-break of this insect. Thereby, the current results may improve the ability of decision supporting systems for the management of this pest by providing better forecasting plans for plant protection managers and pest advisors. And then provide valuable information to plant protection workers in decision-making strategies for pest control. In addition, the underlying mechanism of diapause in relation to environmental cues, such as temperature, may help improve the synchronization between the release of parasitoid wasps and the pest occurrence in the biological control techniques.

## Materials and Methods

### Experimental Insect Culture

In the present study, as the cabbage butterfly *P. melete* is a serious pest of crucifers in the mountain areas of Jiangxi Province, therefore, no specific permits were required for the described insect collecting.

The cabbage butterfly *P. melete* used in this study originated from a population in the vegetable gardens in the suburbs of Nanchang (28°46′N, 115°50′E; at an altitude of between 120–200 m), Jiangxi Province, P. R. China. Full-grown larvae prior to pupation were collected from wild crucifers in late November and were transferred to wooden cages (30×30×35 cm) to pupate and eclose into adults under natural conditions [12].

### Effect of Chilling on Summer and Winter Diapause Development

To induce summer diapause, adults from the overwintering pupae were released to an outdoor web-screened insectary equipped with potted Chinese cabbage, *Brassica chinensis*, for mating and oviposition. Almost all individuals in the first spring generation enter summer diapause. In this experiment, the spring-summer generation larvae were reared from the egg stage in the web-screened insectary at a daily mean temperature of 18.1°C and an increasing day-length from 13 h 9 min to 13 h 50 min. Under these conditions, 99.29% pupae entered summer diapause. To obtain winter diapausing pupae, larvae were reared in outdoor web-screened insectary under a daily mean temperature of 15.0°C with decreasing day-length from 11 h 53 min∼11 h 17 min in late autumn. Under these conditions, 100% pupae entered winter diapause.

Ten days after pupation, diapausing pupae were divided into three groups. Summer and winter diapausing pupae in the first group (control) were held constantly at 20°C and LD 12.5∶11.5 (light 12.5 h: dark 11.5 h) (as control without chilling). The other two groups were transferred to chilling temperatures of 1, 5, 10 or 15°C for different periods of cold treatment, and were then transferred to 20°C and LD12.5∶11.5 in an artificial climate cabinet. Under these conditions, it is helpful for us to investigate the effect of low temperature exposure on diapause development in both summer and winter diapause pupae. Individuals of pupae terminating diapause (i.e. adult emergence) were monitored and recorded daily. The criterion for diapause termination was butterfly eclosion, and the duration of diapause included the period of post-diapause development.

Chilling treatments of 1°C and 5°C combined with continuous darkness (DD) were conducted in a refrigerator (SΛMSUNG BCD-270 FBNW, Suzhou Samsung Electronics Co. Ltd., Suzhou). Cold treatment of 10°C and 15°C under continuous darkness (DD) were conducted in an incubator (QHX-250BS-III, XinMiao Medical Instrument Manufacturer of Shanghai, Shanghai). The pupae were transferred to a round plastic box (15 cm in diameter, 10 cm height), and were kept at 20°C and LD12.5∶11.5 in an incubator, except for the period of cold treatment. The incubator was equipped with four fluorescent 30 W tubes. The light intensity was about 1.97 W·m^2^, variation of temperatures was ±0.5°C, and relative humidity was about 70%. The scotophase was controlled manually by enclosing the containers in opaque hoods.

### Statistical Analysis

Statistical analyses were conducted using the STATA package Version 9.0. Kruskal–Wallis ANOVA tests, followed by Mann-Whitney U test with sequential Bonferroni *posthoc* tests were used to determine whether differences in the duration of diapause among different treatments were significant at α = 0.05. Differences are considered significant if *P*<0.05.

## Supporting Information

Figure S1 **Cumulative eclosion in summer diapausing pupae of** ***P. melete*** **.** The diapausing pupae were transferred to LD12.5∶11.5 at 20°C after exposure to 1°C and DD for different days. The hatched bar indicates the period of cold exposure. Values followed by different letters are significantly different by Bonferroni test (P<0.05).
(TIF)

Figure S2 **Cumulative eclosion in summer diapausing pupae of** ***P. melete.*** The diapausing pupae were transferred to LD12.5∶11.5 at 20°C after exposure to 10°C and DD for different days. The hatched bar indicates the period of cold exposure. Values followed by different letters are significantly different by Bonferroni test (P<0.05).
(TIF)

Figure S3 **Cumulative eclosion in summer diapausing pupae of** ***P. melete*** **.** The diapausing pupae were transferred to LD12.5∶11.5 at 20°C after exposure to 15°C and DD for different days. The hatched bar indicates the period of cold exposure. Values followed by different letters are significantly different by Bonferroni test (P<0.05).
(TIF)

Figure S4 **Cumulative eclosion in winter diapausing pupae of** ***P. melete*** **.** The diapausing pupae were transferred to LD12.5∶11.5 at 20°C after exposure to 1°C and DD for different days. The hatched bar indicates the period of cold exposure. Values followed by different letters are significantly different by Bonferroni test (P<0.05).
(TIF)

Figure S5 **Cumulative eclosion in winter diapausing pupae of** ***P. melete*** **.** The diapausing pupae were transferred to LD12.5∶11.5 at 20°C after exposure to 5°C and DD for different days. The hatched bar indicates the period of cold exposure. Values followed by different letters are significantly different by Bonferroni test (P<0.05).
(TIF)

Figure S6 **Cumulative eclosion in winter diapausing pupae of** ***P. melete*** **.** The diapausing pupae were transferred to LD12.5∶11.5 at 20°C after exposure to 10°C and DD for different days. The hatched bar indicates the period of cold exposure. Values followed by different letters are significantly different by Bonferroni test (P<0.05).
(TIF)

Figure S7 **Cumulative eclosion in winter diapausing pupae of** ***P. melete*** **.** The diapausing pupae were transferred to LD12.5∶11.5 at 20°C after exposure to 15°C and DD for different days. The hatched bar indicates the period of cold exposure. Values followed by different letters are significantly different by Bonferroni test (P<0.05).
(TIF)
