# Supplementary figures and images for: Optimal Low Temperature and Chilling Period for Both Summer and Winter Diapause Development in Pieris melete: Based on a Similar Mechanism
Source: PLoS One. 2013 Feb 18;8(2):e56404. doi: 10.1371/journal.pone.0056404 (PMC3575341; doi:10.1371/journal.pone.0056404)

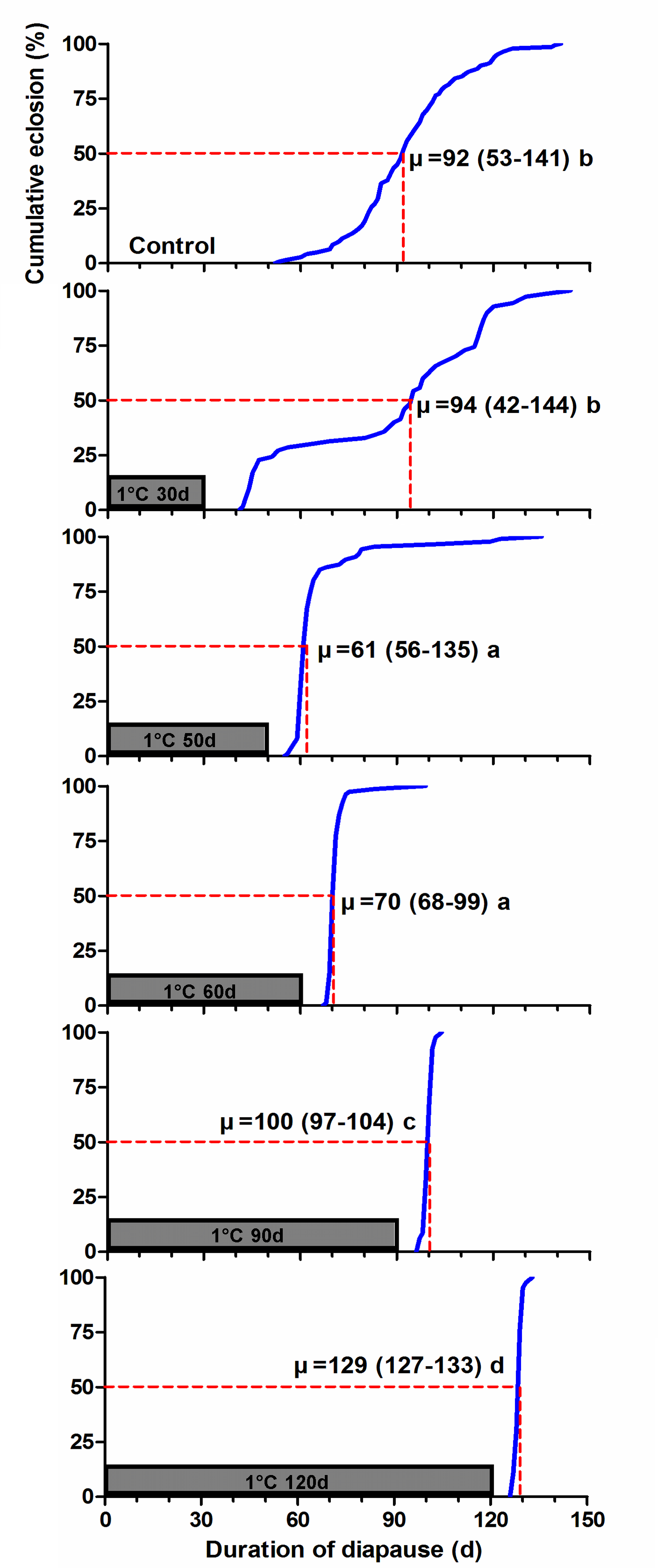

Supplement: Figure S1 — Cumulative eclosion in summer diapausing pupae of P. melete . The diapausing pupae were transferred to LD12.5∶11.5 at 20°C after exposure to 1°C and DD for different days. The hatched bar indicates the period of cold exposure. Values followed by different letters are significantly different by Bonferroni test (P<0.05). (TIF) [file pone.0056404.s001.tif]

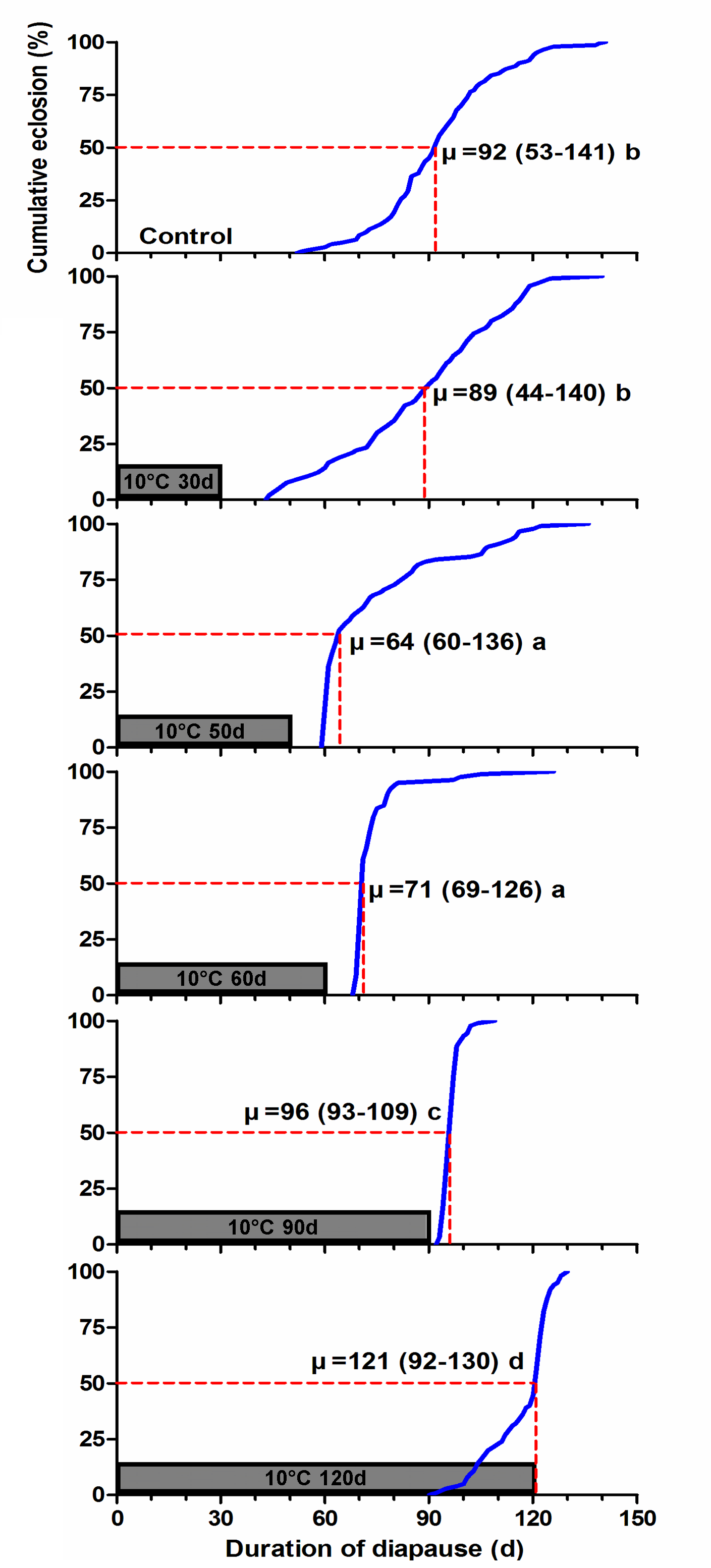

Supplement: Figure S2 — Cumulative eclosion in summer diapausing pupae of P. melete. The diapausing pupae were transferred to LD12.5∶11.5 at 20°C after exposure to 10°C and DD for different days. The hatched bar indicates the period of cold exposure. Values followed by different letters are significantly different by Bonferroni test (P<0.05). (TIF) [file pone.0056404.s002.tif]

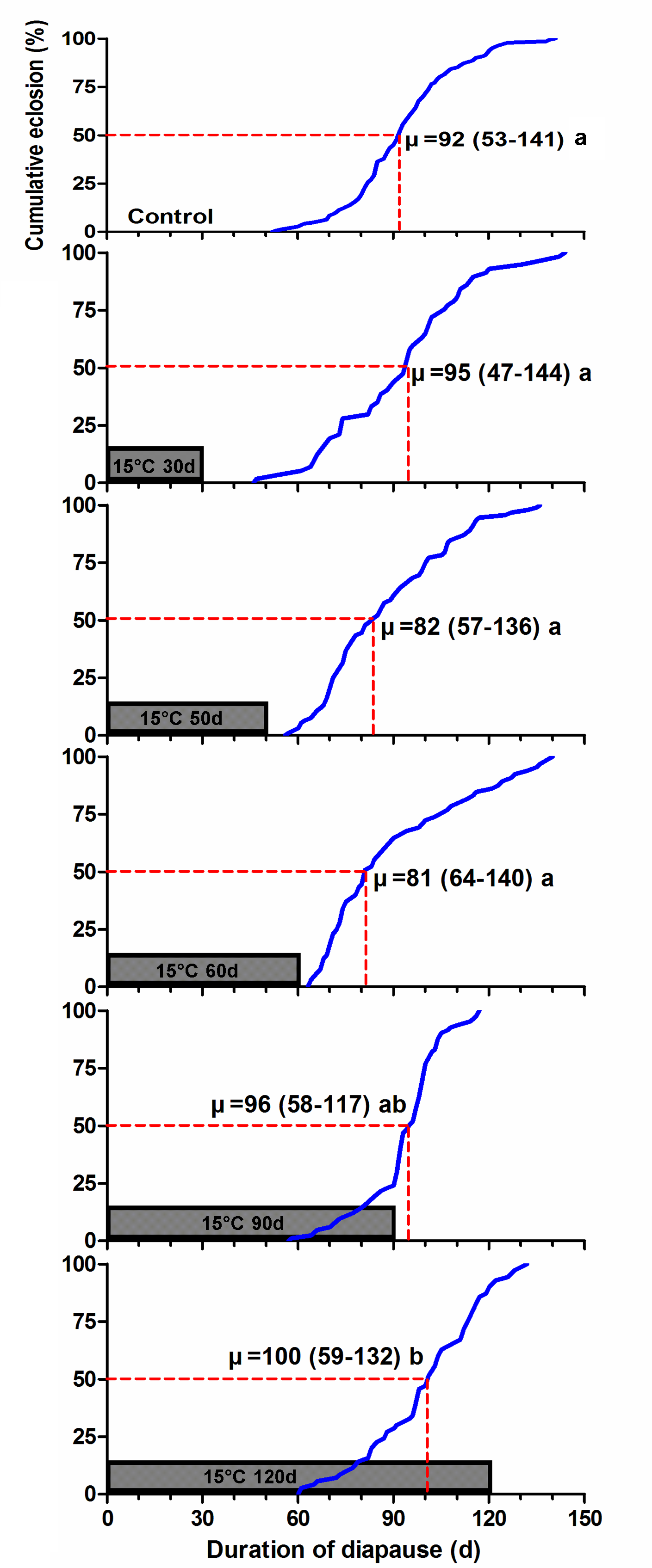

Supplement: Figure S3 — Cumulative eclosion in summer diapausing pupae of P. melete . The diapausing pupae were transferred to LD12.5∶11.5 at 20°C after exposure to 15°C and DD for different days. The hatched bar indicates the period of cold exposure. Values followed by different letters are significantly different by Bonferroni test (P<0.05). (TIF) [file pone.0056404.s003.tif]

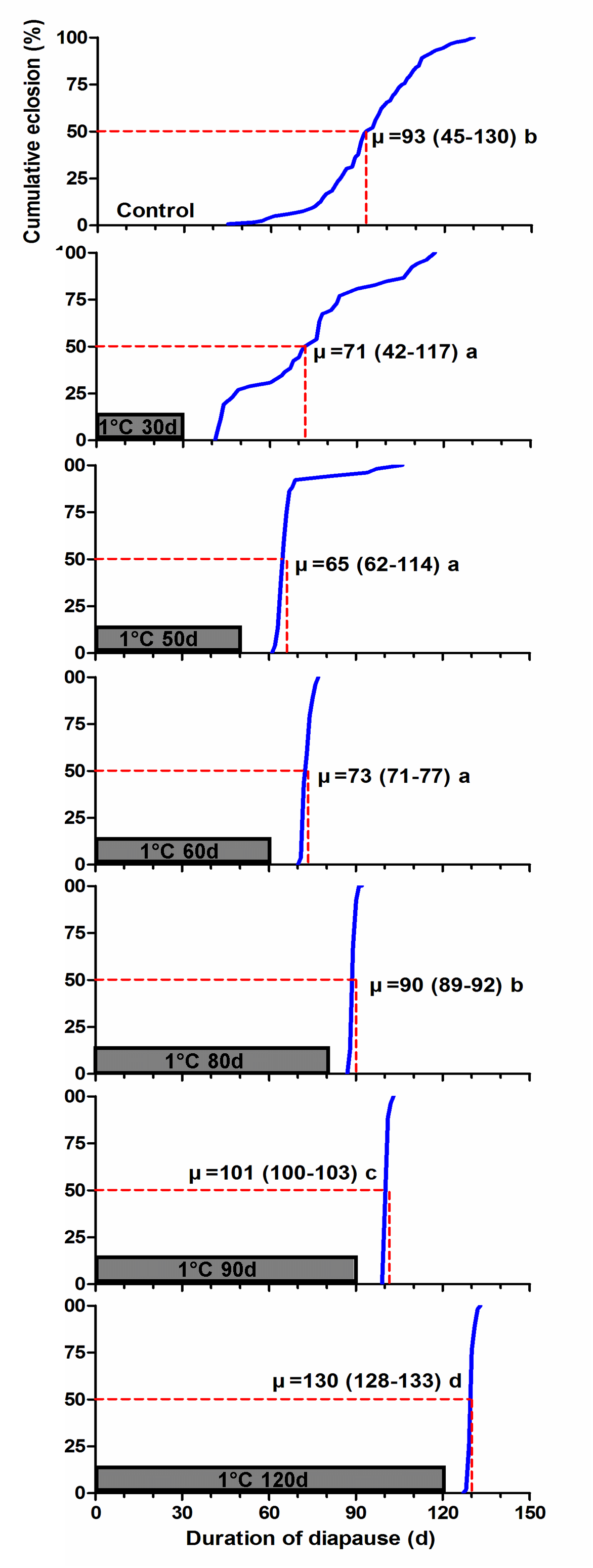

Supplement: Figure S4 — Cumulative eclosion in winter diapausing pupae of P. melete . The diapausing pupae were transferred to LD12.5∶11.5 at 20°C after exposure to 1°C and DD for different days. The hatched bar indicates the period of cold exposure. Values followed by different letters are significantly different by Bonferroni test (P<0.05). (TIF) [file pone.0056404.s004.tif]

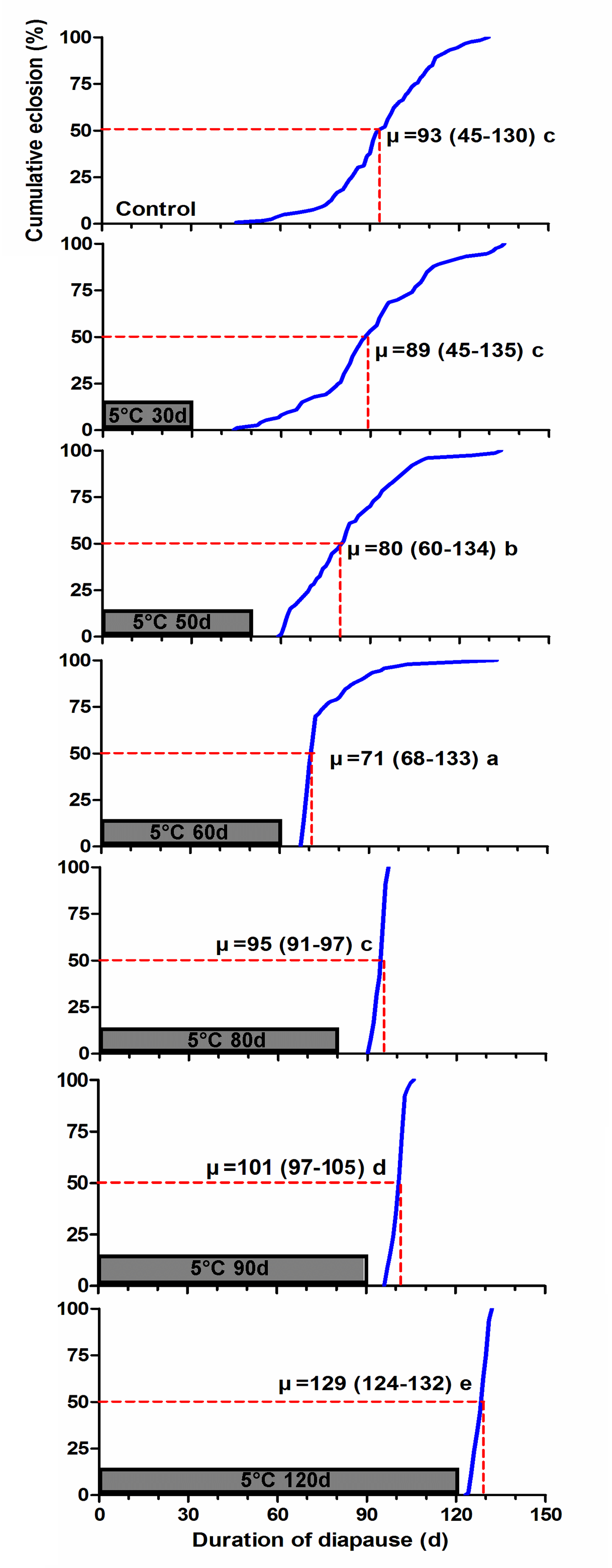

Supplement: Figure S5 — Cumulative eclosion in winter diapausing pupae of P. melete . The diapausing pupae were transferred to LD12.5∶11.5 at 20°C after exposure to 5°C and DD for different days. The hatched bar indicates the period of cold exposure. Values followed by different letters are significantly different by Bonferroni test (P<0.05). (TIF) [file pone.0056404.s005.tif]

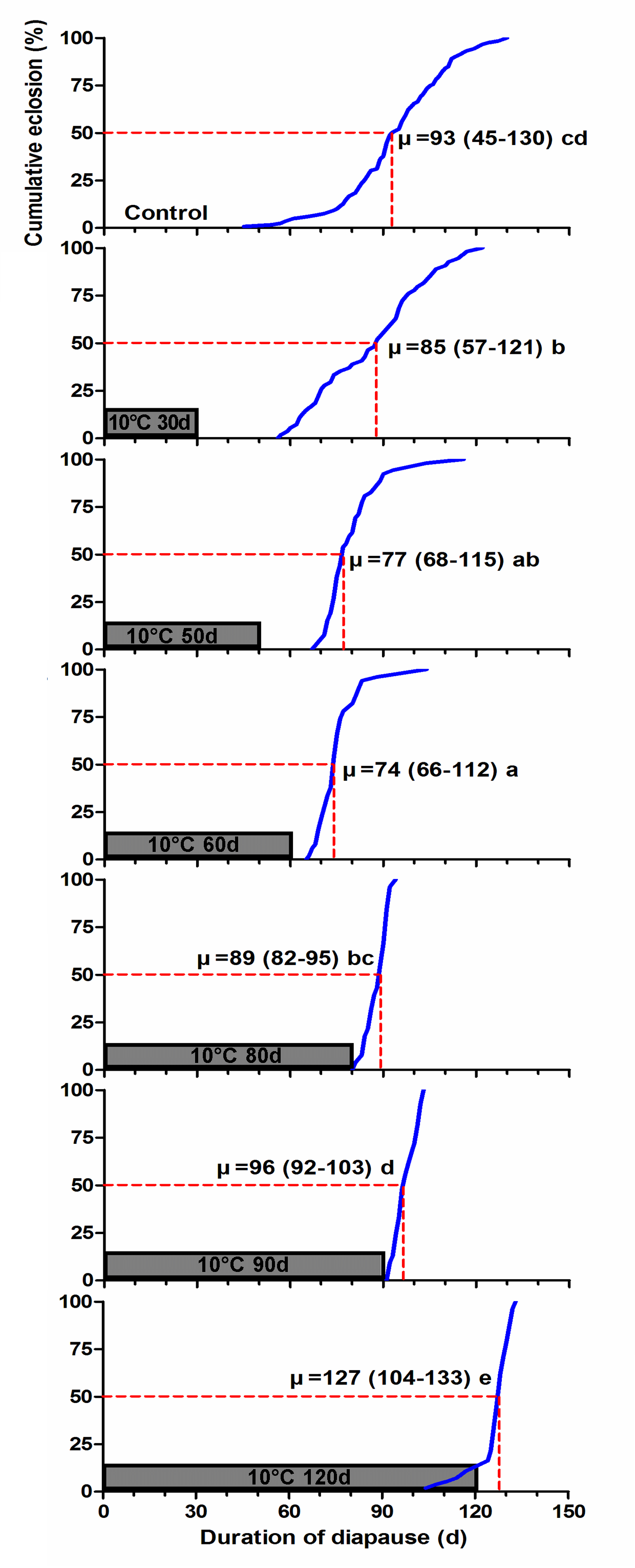

Supplement: Figure S6 — Cumulative eclosion in winter diapausing pupae of P. melete . The diapausing pupae were transferred to LD12.5∶11.5 at 20°C after exposure to 10°C and DD for different days. The hatched bar indicates the period of cold exposure. Values followed by different letters are significantly different by Bonferroni test (P<0.05). (TIF) [file pone.0056404.s006.tif]

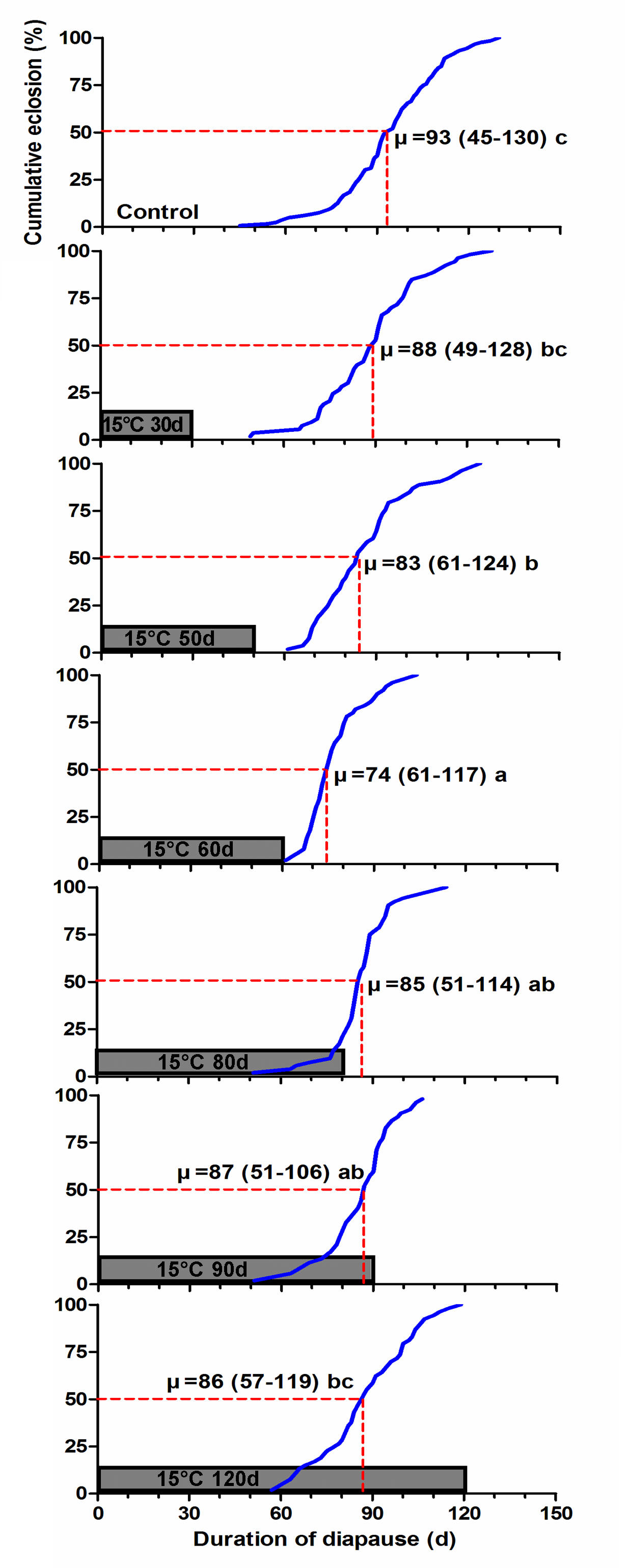

Supplement: Figure S7 — Cumulative eclosion in winter diapausing pupae of P. melete . The diapausing pupae were transferred to LD12.5∶11.5 at 20°C after exposure to 15°C and DD for different days. The hatched bar indicates the period of cold exposure. Values followed by different letters are significantly different by Bonferroni test (P<0.05). (TIF) [file pone.0056404.s007.tif]
